# How and What Do Women Learn About Contraception? A Latent Class Analysis of Adolescents and Adult Women in Delaware

**DOI:** 10.1089/whr.2024.0064

**Published:** 2025-01-28

**Authors:** Mónica L. Caudillo, Fatima Zahra, Michael S. Rendall

**Affiliations:** ^1^ Department of Sociology and Maryland Population Research Center, University of Maryland, College Park, Maryland, USA.; ^2^ GIRL Center, Population Council, Washington, DC, USA.

**Keywords:** contraception, contraceptive information, contraceptive education, adolescents, adult women, reproductive age

## Abstract

**Background:**

Across the reproductive life course, women receive information about contraception that may influence their contraceptive behaviors. This study examines the information sources that adolescents and older women combine to acquire information about contraception.

**Methods:**

A state-representative survey of women aged 18–44 residing in Delaware, US, in 2017 asked from what sources respondents recently learned about contraception and the type of information obtained. The 2017 Delaware Youth Risk Behavior Survey, representative of public high school students aged 14–18, included analogous questions. Latent class analysis was applied to classify respondents in both samples of adolescents (*n* = 1253) and adult women (*n* = 1008) according to the information sources they combined. We estimated multinomial logistic regressions to assess the demographic and reproductive history predictors of using each of the information source repertoires and binomial logistic regressions to analyze their relationship to the information acquired.

**Results:**

Adolescents are more likely than adults to report having recently acquired any information about contraception (76% vs. 64%), but they are more likely to rely primarily on a single source. In contrast, adult women are more likely to combine multiple sources. Age, education, and sexual activity emerged as important predictors of information source repertoires. Adults who combine information sources and adolescents who learn mainly from health care providers or school personnel report the greatest breadth in the contraception-related information acquired.

**Conclusion:**

Interventions to provide or improve contraceptive knowledge may be more effective if they account for how women use and combine information sources, particularly at different stages of their reproductive lives.

## Introduction 

Information acquisition, including both factual knowledge and subjective beliefs, is a critical component of sexual and reproductive health behaviors.^1,2^ Across the reproductive life course, women receive information about contraception that may influence their decision to start or discontinue contraceptive use, or switch to a new method,^3^ which ultimately has consequences for their reproductive health. Furthermore, access to sufficient information about a wide range of contraceptive methods is a precondition for contraceptive autonomy.^4^ Previous research has primarily focused on identifying information sources one by one, and on assessing the demographic predictors of their use on samples restricted to teenagers or young women.^5–9^ For instance, earlier onset of sexual activity and experience of unintended pregnancy has been associated with more learning about contraception from health care providers (HPs),^5^ and racial/ethnic minorities have been found to rely more heavily on information from personal networks.^3,10^ However, in practice, individuals may receive health-related information from multiple and diverse channels at any point in their reproductive lives, and the composition of such source repertoires may shape the quality and content of the information received.^11,12^ For example, while some adolescents may supplement their formal sex education with information from parents,^12^ older women may prefer to combine health information from sources such as doctors and specialized websites,^13–15^ all of which may be associated with varying quality and quantity of information regarding the promotion, effectiveness, and use of contraceptive methods.^16–19^

Although understanding contraceptive information-acquisition processes is key to inform medical practice and public health policy, we know little about the different *combinations* of sources from which women learn about contraception, particularly beyond adolescence. Similarly, the relationship between such source repertoires and the type of information acquired is still poorly understood, and the available evidence is based on small or nonrepresentative samples. In this study, we address this gap in the literature by using two representative samples of adolescent and adult women in Delaware, United States, to identify the diverse combinations or repertoires of contraceptive information sources they use. Next, we evaluate the demographic predictors of the source repertoires that we identify in each of the samples. Finally, we examine how each combination of sources is associated with the type of information received by adolescent and adult respondents.

## Materials and Methods

### Data and samples

We use the 2017 Delaware Youth Risk Behavior Survey (DE YRBS), a cross-sectional probability sample of students enrolled in grades 9–12 in public high schools in the state. The survey was coordinated by the Centers for Disease Control (CDC) and conducted during the spring of 2017.^20,21^ To obtain information about older women, we use data from the Survey of Women (SoW), designed by this research team and fielded from November 2016 to March 2017 by the National Opinion Research Center (NORC) at the University of Chicago.^22^ It is a probability sample of 2873 women aged 18–44 residing in Delaware and Maryland households. For the present analysis, we focused on the 1456 Delaware residents in the sample to preserve comparability with the DE YRBS. We further excluded women who were sterile or infertile (15%) and who had missing values in parity, pregnancy status, and information sources^a^ (15%), leading to an analytical sample of 1008 women. Out of 1483 female adolescents interviewed by the DE YRBS, we restricted the sample to 1253 respondents after excluding those who had missing values in information sources, race, mother’s education, pregnancy history, or sexual activity (15%). We use probability weights in both surveys to obtain estimates that are representative of the household population of women aged 18–44, and public high school female students aged 14–18 in Delaware.^b^

Both surveys include two nearly identical questions on acquisition of contraception-related information. Respondents in the SoW were asked, “In the past 3 months, have you received any information about birth control methods from any of the following places?” They were given a “Yes/No” response option for each of these 10 categories: (1) a friend or family member; (2) Twitter, Facebook, or Snapchat; (3) other social media and internet sources^c^; (4) posters, signs, or billboards; (5) TV or radio; (6) ads or campaigns in community spaces; (7) print ads; (8) nurse, doctor, or other HP; (9) social worker or community health worker; and (10) any other place, with a space to write an answer. Survey respondents could select multiple responses in this question. We created an additional category for learning from school or the workplace, which was based on written responses to the “any other place” option. The DE YRBS included the same question and response options, with the exception that option nine was replaced with school sources, including health teachers, counselors, or other school personnel; option one included sexual partners, in addition to friends and family; and option eight included both HPs—such as nurses and doctors—and social workers outside the school. The DE YRBS also collapsed response options 2 and 3 into “Twitter, Facebook, Instagram, or Snapchat, or other internet sources”; and options 5 and 7 were combined to encompass TV, radio, and print ads.

A second outcome of interest is the type of information acquired. In the SoW, respondents who checked “Yes” to having used at least one source of contraception-related information in the last 3 months were asked, “What types of information have you learned from these sources? (Please check all that apply)” Options were: (1) where you can go to get birth control; (2) how much different birth control methods cost; (3) what types of birth control methods are the most effective at preventing pregnancy; and (4) information about a particular birth control method, such as how it is placed or how it works. The DE YRBS included an analogous question^d^ and the same response options. Respondents were asked to mark all applicable options in both surveys. See Supplementary Appendix Data S1 for printouts of how these questions appeared in each survey.

### Statistical analysis

Latent class analysis (LCA) uses multiple observed variables to predict unobserved subgroups (or classes) that yield clusters of observations that are similar within each class, and distinct from other classes.^25^ This modeling approach uses maximum likelihood to model underlying probability distributions present in the dataset and estimates the person-specific probabilities of belonging to each class. LCA estimates membership in discrete latent groups, which identify distinct combinations of information sources used by respondents.^26^ To identify these classes, we restricted the original datasets to respondents who reported they had learned about contraception from at least one source in the last 3 months in each survey. All the information sources listed in Table 1 were included in the LCA models.

**Table 1.** Summary Statistics of Single Information Sources and Type of Information Acquired Among Adolescent and Adult Women Who Reported Learning About Contraception in the Last 3 Months, 2017 DE YRBS and DE SoW

	DE SoW(Ages 18–44)	DE YRBS(Ages 14–18)	
	%	%	Contrast
Acquired information from this source in last 3 months			
A friend or family member^a^	24.2	43.8	^***^
Social media or other internet sources^b^	23.0	15.3	^***^
Posters, signs, or billboards	16.5	8.3	^***^
TV, radio, or print ads^c^	37.5	13.2	^***^
TV or radio	25.5	—	
Print ads	28.0	—	
Ads or campaigns in the community	8.4	4.9	^*^
A nurse, doctor, or other health care provider	43.0	30.8	^***^
Social or community health worker^d^	1.6	—	
Workplace or school	0.5	—	
Health teacher, school counselor, or other school personnel	—	40.9	
At least one source	63.9	76.0	^***^
Observations	1008	1253	
Type of information acquired (if any source was reported)			
Where you can go to get birth control	37.0	63.9	^***^
How much birth control costs	14.6	26.6	^***^
What birth control methods are the most effective	45.1	52.1	^*^
How a method is placed or how it works	68.0	53.4	^***^
Observations	630	921	

Some labels have been abbreviated. The appendix shows response options as they appeared in questionnaires. Weighted *t*-tests were conducted for comparable variables across surveys.

^*^ *p* < 0.05.

^***^ *p* < 0.001.

^a^ Includes “sexual partners” in the YRBS questionnaire.

^b^ Appears in the YRBS as “Twitter, Facebook, Instagram, or Snapchat or other internet sources.” In the SoW, we combined response categories “Twitter, Facebook or Snapchat” and “Other social media, online advertisements, Google, or other internet sources.”

^c^ Includes respondents who checked “TV or radio” or “Print ads” in the SoW questionnaire. These sources appeared combined in the same response option in the YRBS questionnaire.

^d^ In the YRBS, this response option includes “social worker outside of school.”

DE SoW, Delaware Survey of Women; DE YRBS, Delaware Youth Risk Behavior Survey.

We analyzed adolescents and adults separately to determine the optimal number of latent classes in MPlus 7, starting with a 2-class model. The sample-size adjusted Bayesian Information Criterion (BIC) and the Lo-Mendell-Rubin (LMR) Adjusted Likelihood Ratio Test are recommended in the literature as appropriate tools to decide on the number of latent classes.^27^ We used these goodness of fit indicators along with considerations of theoretical interpretation to select the number of classes that better illustrated the underlying source repertoires for each group. When evaluating the types of sources used by each class, we interpreted family, friends, or partners as “networks;” TV, radio, print ads, posters, and billboards were conceptualized as “traditional media.” The term “health care providers” was applied to nurses and doctors. And for teenagers in the DE YRBS, sources like health teacher, school counselor, or other school personnel were conceptualized as “school” sources. To characterize the source repertoires identified by the LCA, we looked at what defined the distinct classes in each sample: whether respondents in each class relied more heavily on a single type of source, as described above, or a particular combination of sources. Respondents were assigned to classes representing information source repertoires based on the person-specific posterior class categorizations estimated by MPlus 7.

We estimated multinomial logistic models using a series of demographic and reproductive history characteristics as predictors of each information source repertoire, relative to not learning from any source in the last 3 months. Such predictors included age and race for both SoW and YRBS; respondent’s education in the SoW and maternal education in the YRBS; whether the respondent had ever had children in the SoW, and whether they had ever been pregnant in the YRBS. In the SoW, we included a measure of whether the respondent was either: 1) pregnant or trying to get pregnant, 2) sexually active in the last 3 months, but not trying to get pregnant, or 3) not sexually active in the last 3 months. For adolescents in the DE YRBS, we tried to match this predictor as closely as possible and controlled for whether the respondent 1) had never had sex, 2) had ever had sex but were not sexually active in the last 3 months, or 3) had been sexually active in the last 3 months. Sexual activity was defined as having sexual intercourse in both surveys.^e^ Finally, we added foreign-born status as a covariate in the SoW. To assess how source repertoires relate to the information obtained, we estimated bivariate binomial logistic regressions using information source repertoires to predict acquiring each of four different types of content: where to get contraception, cost, effectiveness, or how a method works. Descriptive statistics for all covariates used in these models are shown in Supplementary Appendix Table S1 for the DE YRBS and Supplementary Appendix Table S2 for the SoW. Analyses other than the LCA were conducted in Stata 17.

## Results

### Single information sources

Table 1 presents summary statistics of the information sources and learned content reported in both surveys, showing that 76% of adolescents and 64% of adult women received contraceptive information from at least one of the listed sources in the last 3 months. The use of single sources varied widely between the two age groups, with 38% of adult women learning from traditional media, such as TV, radio or print ads, and 43% learning from HPs, compared with only 13% and 31% of adolescents, respectively. About 41% of adolescents cited learning about contraception in school, while less than 1% of older women mentioned school or workplace sources. Furthermore, whereas 44% of adolescents reported learning from social networks such as friends or family, only 24% of adults cited similar sources. However, this disparity could be partially explained by the fact that this response option also included “sex partners” in the DE YRBS questionnaire. Surprisingly, internet sources were more likely to be used by adult women compared with adolescents (23% vs. 15%). Overall, traditional media and HPs were the most frequently used sources for adult women, and personal networks and school were the most prevalent information sources among adolescents. Regarding the type of information acquired, adult women were more likely than adolescents to learn how a birth control method works (68% vs. 53%), whereas adolescents were more likely to get information about where to get contraception, their cost, and their effectiveness. All these contrasts between age groups were statistically significant (*p* < 0.05).

### Latent class analysis

The sample-size adjusted BIC and the LMR Adjusted Likelihood Ratio Test compare a model with *k* classes to a model with *k* − 1 groups (Supplementary Appendix Table S3 in the Supplementary Appendix Data S1), and were used along with theoretical considerations to decide on the appropriate number of classes for each age group. A smaller BIC and a statistically significant *p* value in the LMR test indicate a better fit compared with the *k* − 1 class model.

A comparison of the sample-adjusted BIC across latent class models for adult women in the SoW revealed that the fit improves progressively as groups increase from two to four but starts worsening when a fifth class is added. The LMR test does not render statistically significant results for any of the comparisons between models of different class numbers, so it does not contribute useful information for this sample. Based on the BIC and given that the four-class model results in groups of distinct theoretical meaning and sample sizes that are adequate for statistical analysis, we concluded that the four-class model is the most appropriate for adult women. For adolescent girls, the BIC suggests improving model fit as the number of classes grows from two to six. However, the LMR test ceases to be significant when comparing the models with five and six classes, which suggests that five classes are preferable to six.^27^ Unfortunately, the five-class model renders a fifth category that is too small for meaningful statistical analysis, with only 29 respondents. Furthermore, exploratory analyses revealed that the information source use of this fifth category is very similar to another group already identified in the four-class model (the “Multiple Sources” repertoire), and therefore not sufficiently distinct on theoretical grounds. For these reasons, we determined that four classes were also optimal for adolescents in the YRBS.

To better understand the predominant information source repertoires in each class, Table 2 shows the distribution of latent classes for each age group. Among adolescent girls, one class acquired information about contraception primarily from their school, while another one learned mainly from their personal networks. Almost a third of respondents combined several sources, such as their HP, personal networks, and school, and a quarter of respondents combined a wider variety of sources. The latter group was characterized by having a moderate-to-high probability of using all the listed sources. It should be noted that schools were a relevant source across all classes of teenagers. Even among respondents who relied primarily on personal networks, about a third complemented their information repertoire with school sources.

**Table 2.** Latent Classes of Information Source Repertoires for Adolescent and Adult Women Who Reported Learning About Contraception in the Last 3 Months, 2017 DE YRBS and SoW

Repertoires	DE YRBS(Ages 14–18)		Repertoires	DE SoW(Ages 18–44)	
	%	n		%	n
Multiple sources	24.9	260	Multiple sources	13.5	72
HP, networks, school	30.7	271	HP	32.0	188
Networks	25.6	245	Networks, internet, HP	28.7	177
School	18.7	155	Traditional media	25.8	193
Total	100.0	931		100.0	630

Samples exclude respondents who did not acquire information from any source in the last 3 months.

HP, health care provider.

In contrast, adult women exhibited a different set of information repertoires. About a third of adult women who received contraceptive information were classified as having learned mainly from an HP. Similar proportions of women acquired information primarily from traditional media, and from a combination of personal networks and internet sources complemented by HPs. A minority of adult women (14%) received contraceptive information from a combination of nearly all sources. The probabilities of using each information source by class in the YRBS and the SoW are shown in Supplementary Appendix Figure S1 and Supplementary Appendix Figure S2 in the Supplementary Appendix Data S1. Exploratory analyses splitting adult women into two groups, (18–30) and (31–44), did not yield differences in results.

### Predictors of information source repertoires

Tables 3 and 4 show relative risk ratios from multinomial logistic regressions that predict information source repertoires for adolescents and older women, respectively. Women who did not receive information about contraception from any source in the last 3 months are the reference outcome category. For adolescents (Table 3), the most relevant predictors of information source repertoires are age and sexual activity, even after controlling for race, maternal education, or having ever been pregnant. Older adolescents have higher odds of acquiring information from an HP, combined with personal networks and school, relative to not learning from any source. In contrast, the relative risk of learning from multiple sources, primarily networks, or primarily school, is not predicted by age among adolescents. Compared with those who have never had sex, those who have experienced sexual onset have higher odds of learning from two repertoires: (1) an HP, combined with personal networks and school, and (2) mainly from personal networks. This is especially the case for adolescents who were sexually active in the last 3 months.

**Table 3.** Multinomial Logistic Regression Predicting Information Source Repertoires (Reference = Did Not Learn about Contraception in Past 3 Months), Adolescent Girls Aged 14–18, DE YRBS

	Multiple sources	HP, networks, school	Networks	School
Age (ref ≤14)				
15	0.92	2.05	1.01	0.94
	[0.34]	[1.04]	[0.37]	[0.33]
16	1.09	3.03^*^	1.21	0.79
	[0.44]	[1.45]	[0.43]	[0.34]
17	1.55	2.62^*^	1.13	0.8
	[0.59]	[1.12]	[0.45]	[0.39]
18+	1.5	3.48^*^	1.37	1.22
	[0.63]	[1.82]	[0.60]	[0.66]
Mother’s education (ref = less than HS)				
High school	0.87	1.41	1.06	1.06
	[0.41]	[0.54]	[0.35]	[0.44]
Some college	1.66	1.14	1.24	0.62
	[0.60]	[0.52]	[0.47]	[0.29]
BA or more	1.25	1.08	1.19	0.8
	[0.49]	[0.42]	[0.34]	[0.26]
Race (ref = White)				
Non-Hispanic Black	0.76	0.75	1.02	1.18
	[0.20]	[0.18]	[0.26]	[0.26]
Asian	1.19	1.29	0.89	0.5
	[0.49]	[0.59]	[0.60]	[0.29]
Non-Hispanic Other	1.39	1.04	0.99	0.38
	[0.72]	[0.62]	[0.59]	[0.22]
Hispanic	1.14	0.68	1.15	1.02
	[0.36]	[0.21]	[0.43]	[0.38]
Sexual activity (ref = never had sex)				
Not sexually active	1.23	2.74^**^	1.43	0.39
	[0.56]	[0.94]	[0.55]	[0.21]
Sexually active	1.32	4.56^***^	2.55^***^	1.81
	[0.30]	[1.12]	[0.59]	[0.61]
Ever pregnant	2.75	1.03	0.34	1.17
	[2.02]	[0.58]	[0.26]	[0.86]
*N*	1253			

Exponentiated coefficients; standard errors in brackets.

^*^ *p* < 0.05.

^**^ *p* < 0.01.

^***^ *p* < 0.001.

**Table 4.** Multinomial Logistic Regression Predicting Contraceptive Information Source Repertoires (Reference = Did Not Learn About Contraception in Past 3 Months), Adult Women Aged 18–44, DE Survey of Women

	Multiple Sources	Health care Provider	Networks, internet, HP	Traditional Media
Age (ref = 18–24)				
25–29	0.39	0.42^*^	0.37^**^	1.1
	[0.21]	[0.17]	[0.14]	[0.47]
30–44	0.20^**^	0.18^***^	0.14^***^	0.65
	[0.11]	[0.07]	[0.05]	[0.27]
Education (ref = HS or less)				
Some college	3.96^*^	2.26^*^	2.96^**^	1.92
	[2.27]	[0.87]	[1.16]	[0.75]
BA or more	3.09	1.78	3.79^***^	2.43^*^
	[1.78]	[0.73]	[1.40]	[0.93]
Race (ref = White)				
Non-Hispanic Black	1.31	1.14	0.68	0.93
	[0.60]	[0.38]	[0.28]	[0.34]
Asian	0.36	2.87	1.28	2.07
	[0.37]	[1.93]	[0.86]	[1.43]
Non-Hispanic Other	1.1	0.42	0.81	0.79
	[0.94]	[0.33]	[0.47]	[0.45]
Hispanic	0.75	0.42	0.66	1.3
	[0.56]	[0.21]	[0.37]	[0.63]
Sexual activity (ref = pregnant/trying)				
Not sexually active	1.69	0.58	0.75	0.73
	[1.17]	[0.31]	[0.34]	[0.33]
Sexually active	1.06	2.11	0.85	0.9
	[0.75]	[0.89]	[0.33]	[0.35]
Foreign born	1.2	0.53	0.59	0.36^*^
	[0.72]	[0.25]	[0.32]	[0.18]
Live births (ref = none)	0.63	0.84	0.71	0.73
One child	[0.32]	[0.28]	[0.25]	[0.26]
	0.49	1.49	0.92	1.31
Two or more children	[0.25]	[0.49]	[0.34]	[0.43]
*N*	1008			

Exponentiated coefficients; standard errors in brackets.

^*^ *p* < 0.05.

^**^ *p* < 0.01.

^***^ *p* < 0.001.

Among adult women (Table 4), even after controlling for socioeconomic and reproductive characteristics, age was *negatively* associated with the relative risk of learning from three out of four source repertoires: (1) multiple sources, (2) mainly HP, and (3) a combination of personal networks, internet, and HP. There was no statistically significant association between age and the odds of learning mainly from traditional media, relative to not learning from any source. Having at least some college was associated with higher odds of acquiring information about contraception from any source repertoire in the last 3 months, compared with having no more than high school. Race/ethnicity, sexual activity, and parity were not significantly associated with information source repertoires. Being foreign born was negatively associated with learning primarily from traditional media, but otherwise no different from U.S.-born women.

### Source repertoires and acquired information

**FIG. 1.**
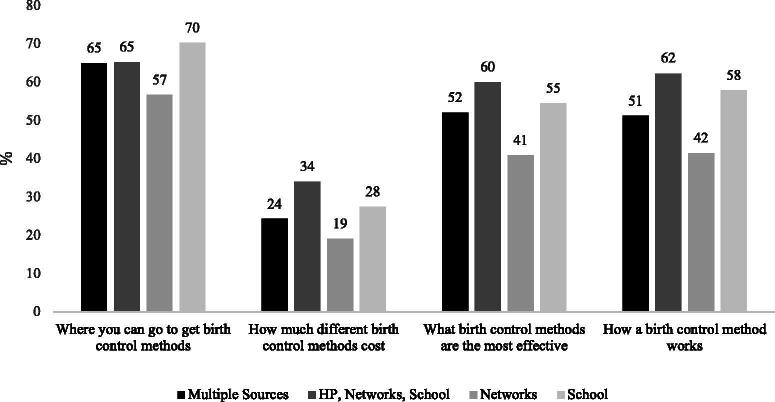
Probability of acquiring different types of information by source repertoire in Delaware, adolescent girls aged 14–18, 2017 DE YRBS. Sample excludes respondents who did not acquire information from any source in the last 3 months in the analytical sample, and 10 additional respondents who reported learning from an information source but did not answer the question about acquired content. *N* = 921. DE YRBS, Delaware Youth Risk Behavior Survey; HP, health care provider.

**FIG. 2.**
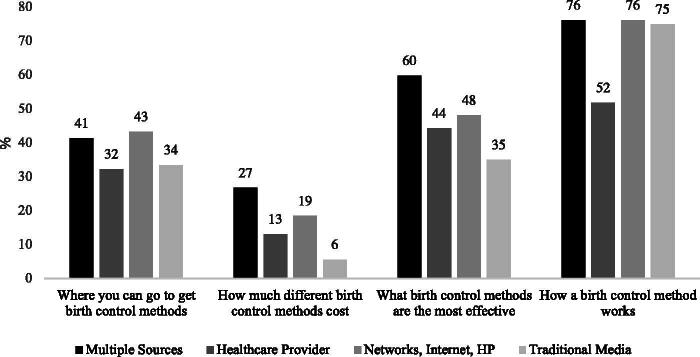
Probability of acquiring different types of information by source repertoire in Delaware, adult women aged 18–44, 2017 DE SoW. Sample excludes respondents who did not acquire information from any source in the last 3 months in the analytical sample. *N* = 630. HP, health care provider; DE SoW, Delaware Survey of Women.

Figures 1 and 2 show predicted probabilities from bivariate binary logistic regressions using the source repertoires identified by our LCA to predict each of the four types of information content acquired by adolescents and adult women, respectively. Adolescents who obtain information primarily from personal networks are less likely to learn about a variety of topics related to contraceptives (where to get them, how much they cost, their effectiveness, and how they work) relative to those who receive contraceptive information mainly from school; from a combination of HPs, personal networks, and school; or from multiple sources. Formal comparisons of probabilities (see Supplementary Appendix Table S4 in the Supplementary Appendix Data S1) show that adolescents who obtained information from source repertoires that drew heavily from institutionalized sources, such as the school or an HP, learned about the broadest array of contraception-related information topics, and had higher probabilities of obtaining technical details.

In contrast to teenagers, for whom using mainly institutionalized sources was associated with the greatest information acquisition, it is adult women who combine sources who have the highest probability of learning about contraception (Fig. 2). Notably, adult women who learn mainly from HPs are disadvantaged when it comes to acquiring information about how a method works, relative to those who had more diversified source repertoires, such as the combination of multiple sources, or of personal networks, the internet, and an HP (Fig. 2), and these differences are statistically significant (Supplementary Appendix Table S4).

In addition, adult women who learned mainly from traditional media (such as TV, radio, and print ads) had statistically significantly lower probabilities of learning about the cost and effectiveness of contraception compared with those who combined multiple sources. They also had a lower probability of acquiring information about how much contraception costs relative to women who learned from a combination of personal networks, the internet, and an HP. Such contrast was statistically significant as well (Supplementary Appendix Table S4).

## Discussion

Our study adopted an innovative “information source repertoire” approach to describing women’s experiences of receiving information about contraception from various sources throughout their reproductive lives using two state-representative surveys. In addition to assessing information source repertoires, a key contribution of our article is that it is one of the first studies to analyze contraception information acquisition across the life course,^13^ including both adolescents and adult women through ages 14–44, whereas previous studies have focused mainly on adolescent girls and young women. Furthermore, prior research on contraception-related information acquisition has often relied on nonrepresentative or smaller samples,^2,10,28,29^ while our study uses population-representative data from two surveys in Delaware. Taken together, these contributions extend existing quantitative literature that has only analyzed information sources one by one and build on qualitative studies that have used nonrepresentative samples to document how women use multiple sources to learn about contraception.^2,10,28,29^

Compared with adults, teenagers were overall more likely to report recently acquiring information about contraception. Our results revealed that three out of four of the source repertoires used by adolescents featured school sources prominently. The second information source that was included in most of the repertoires used by adolescents was personal networks, with most respondents in this age group combining it with other sources, and with about a fourth of them relying primarily on personal networks to obtain contraceptive-related information. This echoes previous quantitative and qualitative evidence that family, and specifically older women in the household, can strongly influence adolescent’s contraceptive use and method choice.^2,19,30^ Nonetheless, our analyses also suggest that compared with adolescents who rely heavily on institutionalized sources, such as school or HPs, those who acquired their contraception-related information mainly from personal networks were less likely to learn about how much it costs, where to get it, how effective it is, or how it works. This is consistent with qualitative evidence suggesting that mothers may influence their daughters’ contraceptive behavior by either serving as role models or by helping with the logistics of obtaining certain types of birth control^19^ but not necessarily by acting as sources of medical information. Overall, adolescents who relied heavily on institutionalized sources such as school or HPs were more likely to acquire information about the four types of content that we evaluated (where to get it, effectiveness, cost, or how it works). However, these results should be interpreted as associations between the sources used and the kind of information acquired, since our research design does not allow us to attribute a causal link.

Relative to adolescents, the source repertoires of adult women were more diversified and less heavily dependent on a single source. HPs were included in most repertoires for adult women, either as a primary or supplementary source of contraception-related information. However, in contrast to adolescents, for adult women learning primarily from HPs was associated with a lower probability of learning about how a method works. This is consistent with qualitative evidence on the multiple opportunities for improvement in medical contraceptive counseling.^31^ Alternatively, our results might suggest that as women age, they may be less dependent on HPs to acquire contraception-related information, and more likely to benefit from blending a diverse array of sources. Nonetheless, as mentioned above, we are only able to identify associations between source repertoires and acquired information.

For adolescent girls, age and sexual activity seem to be the most important predictors of recently learning about contraception. However, the relationship with age is a nuanced one. While age is *positively* associated with the probability of learning among adolescents, it is *negatively* associated with the probability of doing so for adult women. These findings suggest that there is a nonlinear relationship between age and contraception-related information acquisition, with adolescents being more likely to acquire information as they age and become sexually active, and women then reducing their likelihood of obtaining more information as they go past age 25. One reason why adolescents are more likely to report recently acquiring information is that they are less knowledgeable and experienced with contraception, and therefore both seek information about it and recall exposure to such information. Another is they may be targeted by sex education programs and other information providers. Related to these findings, a major difference we found between the types of information acquired between the two groups was that adolescents were much more likely than were adult women to obtain logistical information about where to get birth control methods and how much they cost.

Our study faces some limitations. As mentioned earlier, given the nature of cross-sectional data, for example, we cannot ascertain a causal link between sexual and reproductive outcomes and the likelihood of using a given source repertoire. We are also unable to infer from these data whether source repertoires are a result of differences in preferences for sources of information or of differences in access to sources of information. Furthermore, we are unable to distinguish between cases in which respondents sought information from a specific source, as opposed to being passively exposed to it. Lastly, our data do not distinguish which types of information are obtained from which sources, making the link between specific information sources and learned content difficult to establish.

## Conclusions

Our analysis shows that interventions need to be cognizant of the different information source repertoires used by adolescent and adult women, and of the relationship between the information sources they combine and the knowledge they acquire on contraceptive access, effectiveness, and usage. Due to adult women’s tendency to combine multiple sources to obtain information that may not be covered in their medical visits, or that they may not think a medical visit is necessary for, it may be possible to successfully target them with health education campaigns based on complementary channels such as the internet. Nonetheless, it continues to be important to train and support HPs to provide patients with thorough information on contraception. Adolescents may be better served by interventions focused on enhancing the information they obtain from institutionalized sources, such as the school and HPs, given their propensity to learn mainly from one or very few sources, and the strong potential of institutionalized channels to deliver accurate and comprehensive information.

## Notes

^a^
Age, race, education, and nativity, which were imputed by NORC using a hot-deck strategy.

^b^
The YRBS is a representative sample of high school students, and as such, it includes a few (six) respondents who were younger than 14, and 114 students who were 18 or older.

^c^
Our data were collected coincident with a contraceptive access initiative in Delaware from 2015 to 2020.^23^ A component of this initiative was an internet-based provision of information in Delaware to promote reproductive health clinic attendance aimed at, but not restricted to, women aged 18–29.^24^ This component of the intervention, however, started in May 2017, shortly after the data collection of our DE YRBS and SoW samples concluded.^20,22^

^d^
In the DE YRBS, the wording of the main question was: “In the past 3 months, did you receive information from any sources on the following topics?”

^e^
In the SoW, the question asking about sexual activity specifies that it refers to penile-vaginal sex with a male, whereas the YRBS refers more generally to sexual intercourse.

## Abbreviations Used

BIC: Bayesian Information Criterion
DE YRBS: Delaware Youth Risk Behavior Survey
DE SoW: Delaware Survey of Women
HP: Healthcare provider
LCA: Latent Class Analysis
LMR: Lo-Mendell-Rubin
NORC: National Opinion Research Center
