# Supplementary material for: How and What Do Women Learn About Contraception? A Latent Class Analysis of Adolescents and Adult Women in Delaware
Source: Womens Health Rep (New Rochelle). 2025 Jan 28;6(1):136–46. doi: 10.1089/whr.2024.0064 (PMC11839519; doi:10.1089/whr.2024.0064)
Supplement: Supplementary Appendix Data S1 [file whr.2024.0064_supplementary_data.pdf]

Information Source and Content Learned Question Printouts

A. 2017 DE Survey of Women

26 In the past 3 months, have you received any information about birth control methods from any of the following places?

	Yes	No	Don't Know	Prefer not to answer
a. A friend or family member	1☐	2☐	3☐	4☐
b. Twitter, Facebook, or Snapchat	1☐	2☐	3☐	4☐
c. Other social media, online advertisements, Google, or other internet sources	1☐	2☐	3☐	4☐
d. Posters, signs, or billboards	1☐	2☐	3☐	4☐
e. TV or Radio	1☐	2☐	3☐	4☐
f. Ads or campaigns in the community, such as at bars, restaurants, or other local events	1☐	2☐	3☐	4☐
g. Print ads, such as in magazines, newspapers, and brochures	1☐	2☐	3☐	4☐
h. A nurse, doctor, or other healthcare provider	1☐	2☐	3☐	4☐
i. Social worker or community health worker	1☐	2☐	3☐	4☐
j. Any other place, please specify any other place(s): ______	1☐	2☐	3☐	4☐

If you answered No to all items in Question 26, please skip to Question 28. Otherwise, continue to Question 27.

27 What types of information have you learned from these sources? *Please check all that apply.*

1 ☐ Where you can go to get birth control methods
2 ☐ How much different birth control methods cost
3 ☐ What types of birth control methods are the most effective at preventing pregnancy
4 ☐ Information about a particular birth control method, such as how it is placed or how it works
5 ☐ Other information, please specify: ______
6 ☐ Prefer not to answer

B. 2017 DE YRBS

90. In the past 3 months, have you gotten any information about birth control methods from any of the following sources? **(Mark all that apply.)**

- ◯ A friend, family member, or sexual partner
- ◯ Health teacher, school counselor, school wellness center, or other school personnel
- ◯ Twitter, Facebook, Instagram, or Snapchat or other Internet sources
- ◯ Posters, signs, or billboards
- ◯ TV, radio, or print ads, such as in magazines, newspapers, and brochures
- ◯ Ads or campaigns in the community or at local events
- ◯ A nurse, doctor, other healthcare provider or social worker outside of school
- ◯ I have not gotten any information about birth control from any of these sources

91. In the past 3 months did you receive information from any sources on the following topics? **(Mark all that apply.)**

- ◯ Where you can go to get birth control
- ◯ How much birth control costs
- ◯ What types of birth control are the most effective
- ◯ Information about a particular birth control method, such as how it is placed or how it works
- ◯ I have not received any information on these topics from any sources
