# Supplementary material for: How and What Do Women Learn About Contraception? A Latent Class Analysis of Adolescents and Adult Women in Delaware
Source: Womens Health Rep (New Rochelle). 2025 Jan 28;6(1):136–46. doi: 10.1089/whr.2024.0064 (PMC11839519; doi:10.1089/whr.2024.0064)
Supplement: Supplementary Appendix Figure S1 [file whr.2024.0064_supplementary_figuresa1.pdf]

Figure 1A. Probability of Using Single Information Sources for each of Four Latent Class Source Repertoires in Delaware, Adolescent Girls Aged 14-18, 2017 DE YRBS

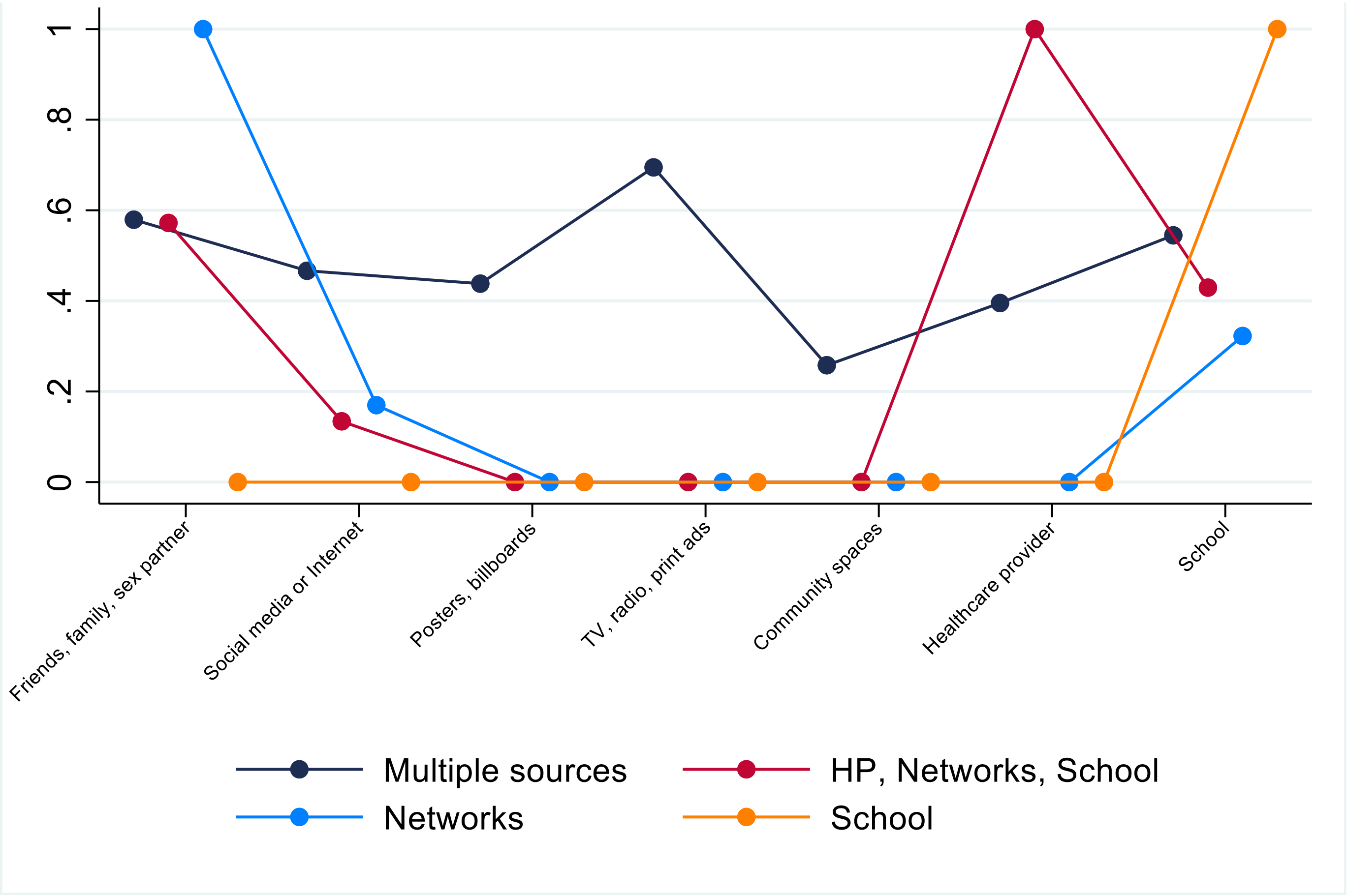

Notes: Sample excludes respondents who did not acquire information from any source in the last 3 months. N = 931. HP = Healthcare provider.
