# Supplementary material for: How and What Do Women Learn About Contraception? A Latent Class Analysis of Adolescents and Adult Women in Delaware
Source: Womens Health Rep (New Rochelle). 2025 Jan 28;6(1):136–46. doi: 10.1089/whr.2024.0064 (PMC11839519; doi:10.1089/whr.2024.0064)
Supplement: Supplementary Appendix Table S1 [file whr.2024.0064_supplementary_tablesa1.pdf]

Table A1. Descriptive statistics of covariates, Delaware Youth Risk Behavior Survey (DE YRBS) 2017

	All Respondents	Did not Learn about Contraception in Past 3 Months	Learned about Contraception in Past 3 Months			
			Multiple Sources	HP, Networks, School	Networks	School
Age						
14	11.7	16.1	12.7	4.2	11.5	15.6
15	22.5	27.0	20.7	18.0	21.8	25.7
16	24.7	23.1	21.4	29.8	25.9	21.6
17	25.8	22.8	30.5	28.6	25.1	21.4
18+	15.2	11.0	14.7	19.4	15.6	15.7
Mother's Education						
Less than HS	14.6	15.0	14.5	13.0	13.6	17.9
High School	22.3	20.8	15.7	28.0	20.9	26.3
Some College	18.5	18.0	23.4	17.8	19.3	12.9
BA or more	44.6	46.2	46.4	41.2	46.2	42.9
Race						
White	47.9	46.9	48.4	51.4	45.8	45.8
Non-Hispanic Black	30.7	32.1	26.2	28.3	32.7	35.5
Asian	4.0	4.2	5.0	4.7	3.3	2.0
Non-Hispanic Other	2.9	2.5	4.1	3.7	2.8	0.9
Hispanic	14.5	14.2	16.3	11.9	15.4	15.8
Sexual activity						
Never had sex	56.2	71.6	61.7	34.4	52.7	63.4
Not sexually active	8.6	8.1	10.0	11.5	8.7	2.9
Sexually active	35.2	20.3	28.2	54.2	38.6	33.7
Ever pregnant	3.0	1.8	5.6	3.9	1.1	3.0
N	1253	322	260	271	245	155

*Respondents who learned about contraception in the last 3 months are broken down by information source repertoire.
