# Supplementary material for: How and What Do Women Learn About Contraception? A Latent Class Analysis of Adolescents and Adult Women in Delaware
Source: Womens Health Rep (New Rochelle). 2025 Jan 28;6(1):136–46. doi: 10.1089/whr.2024.0064 (PMC11839519; doi:10.1089/whr.2024.0064)
Supplement: Supplementary Appendix Table S2 [file whr.2024.0064_supplementary_tablesa2.pdf]

Table A2. Descriptive statistics of covariates, Delaware Survey of Women (DE SoW) 2017

	All Respondents	Did not Learn about Contraception in Past 3 Months	Learned about Contraception in Past 3 Months*			
			Multiple Sources	Healthcare Provider	Networks, Internet, HP	Traditional Media
Age	30.8	16.2	50.3	41.3	49.4	19.1
18-24	22.3	20.2	19.5	23.1	22.8	26.7
25-29	46.9	63.6	30.2	35.6	27.8	54.2
30-44						
Education						
HS or Less	18.1	26	9.3	16.4	11.1	15.3
Some College	44.4	37.2	59.3	51.7	47.7	39.3
BA or more	37.5	36.7	31.4	31.8	41.2	45.3
Race						
White	64.5	61.4	62.2	63.2	71.4	66.5
Non-Hispanic Black	17.8	20.1	21.1	20	11.3	15.6
Asian	6.8	5.6	2.2	11.5	6.5	6.2
Non-Hispanic Other	5.6	6.8	8.9	2.4	6.2	4.4
Hispanic	5.3	6.1	5.6	2.8	4.7	7.2
Sexual activity						
Pregnant/Trying	11.1	12.8	7.5	6.9	12.1	13.2
Not sexually active	23.9	25.5	45	10.6	28.1	21.5
Sexually active	65	61.7	47.4	82.5	59.8	65.3
Foreign born	13.1	17.3	11.8	12.9	8.7	9.7
Live births						
None	54.1	44.1	73.8	54	69.4	48.8
1 child	19.4	24.4	15.5	17.5	15.1	17.8
2 or more children	26.5	31.4	10.7	28.6	15.5	33.4
N	1008	378	72	188	177	193

*Respondents who learned about contraception in the last 3 months are broken down by information source repertoire.
