# Supplementary material for: How and What Do Women Learn About Contraception? A Latent Class Analysis of Adolescents and Adult Women in Delaware
Source: Womens Health Rep (New Rochelle). 2025 Jan 28;6(1):136–46. doi: 10.1089/whr.2024.0064 (PMC11839519; doi:10.1089/whr.2024.0064)
Supplement: Supplementary Appendix Table S3 [file whr.2024.0064_supplementary_tablesa3.pdf]

Table A3. Goodness of Fit Indicators for Nested Latent Class Models with Increasing Class Numbers

	DE YRBS					DE SoW				
**Goodness of Fit Indicators**	**2-class**	**3-class**	**4-class**	**5-class**	**6-class**	**2-class**	**3-class**	**4-class**	**5-class**	**6-class**
Sample-size adjusted BIC	7004.5	6874.6	6818.4	6709.8	6696.0	6083.4	5955.4	5906.0	5915.6	5922.4
Loglikelihood	-3474.1	-3394.1	-3351.0	-3281.7	-3259.8	-3009.4	-2928.4	-2886.7	-2874.5	-2861.0
Lo-Mendell-Rubin Adjusted LR Test	***	**	**	*	N.S.	N.S.	N.S.	N.S.	N.S.	N.S.

Notes: * p<0.05, ** p<0.01, *** p<0.001. N.S. = Non-significant.
