# Supplementary material for: How and What Do Women Learn About Contraception? A Latent Class Analysis of Adolescents and Adult Women in Delaware
Source: Womens Health Rep (New Rochelle). 2025 Jan 28;6(1):136–46. doi: 10.1089/whr.2024.0064 (PMC11839519; doi:10.1089/whr.2024.0064)
Supplement: Supplementary Appendix Table S4 [file whr.2024.0064_supplementary_tablesa4.pdf]

Table A4. Differences Between Information Source Repertoires in Probabilities of Acquiring Each Type of Information, 2017 DE YRBS and DE SoW

Adolescent girls (N=921), 14-18, 2017 DE YRBS

	Where		Cost		Effectiveness		Work	
HP, Networks, School vs Multiple sources	0.2		9.7		7.8		11.1	*
Networks vs Multiple sources	-8.3		-5.1		-11.2	*	-9.7	*
School vs Multiple sources	5.3		3.3		2.4		6.6	
Networks vs HP, Networks, School	-8.5		-14.8	***	-19.1	***	-20.8	***
School vs HP, Networks, School	5.1		-6.4		-5.4		-4.4	
School vs Networks	13.6	*	8.4		13.6	*	16.4	**

Adult women (N=630), 18-44, 2017 DE SoW

	Where		Cost		Effectiveness		Work	
HP vs Multiple sources	-8.9		-13.6		-15.5		-24.3	***
Networks, Internet, HP vs Multiple sources	2		-8.2		-11.7		0	
Traditional Media vs Multiple sources	-7.8		-21	*	-24.8	*	-1.2	
Networks, Internet, HP vs HP	11		5.4		3.8		24.3	***
Traditional Media vs HP	1.1		-7.5		-9.3		23.1	***
Traditional Media vs Networks, Internet, HP	-9.9		-12.9	***	-13.1		-1.2	

Note: Samples excludes respondents who did not learn information from any source in the last 3 months in the analytical sample, and 10 additional respondents who reported learning from an information source but did not answer the question about acquired content in the YRBS.

* $p<0.05$, ** $p<0.01$, *** $p<0.001$
